# Nutritional Treatment in Crohn’s Disease

**DOI:** 10.3390/nu13051628

**Published:** 2021-05-12

**Authors:** Giacomo Caio, Lisa Lungaro, Fabio Caputo, Eleonora Zoli, Fiorella Giancola, Giuseppe Chiarioni, Roberto De Giorgio, Giorgio Zoli

**Affiliations:** 1 Department of Translational Medicine, University of Ferrara, 44121 Ferrara, Italy; lnglsi@unife.it (L.L.); fabio.caputo@unife.it (F.C.); Zolieleonora@yahoo.com (E.Z.); fiorella.giancola2@unibo.it (F.G.); dgrrrt@unife.it (R.D.G.); 2 Center for the Study and Treatment of Chronic Inflammatory Intestinal Diseases (IBD) and Gastroenterological Manifestations of Rare Diseases, Department of Translational Medicine, University of Ferrara, 44121 Ferrara, Italy; 3 Center for the Study and Treatment of Alcohol-Related Diseases, Department of Translational Medicine, University of Ferrara, 44121 Ferrara, Italy; 4 Mucosal Immunology and Biology Research Center, Massachusetts General Hospital-Harvard Medical School, Boston, MA 02114, USA; 5 Department of Internal Medicine, Santissima Annunziata Hospital, Cento (Ferrara), University of Ferrara, 44042 Ferrara, Italy; 6 Division of Gastroenterology of the University of Verona, A.O.U.I. Verona, 37126 Verona, Italy; giuseppe.chiarioni@univr.it; 7 Division of Gastroenterology and Hepatology, University of North Carolina at Chapel Hill, Chapel Hill, NC 27599-7080, USA

**Keywords:** Crohn’s disease, inflammatory bowel disease, enteral nutrition, parenteral nutrition, low FODMAP diet, specific carbohydrate diet, nutrigenomics

## Abstract

Crohn’s disease (CD) is a chronic inflammatory bowel disease (IBD) which can affect any part of the whole gastrointestinal tract (from mouth to anus). Malnutrition affects 65–75% of CD patients, and it is now well acknowledged that diet is of paramount importance in the management of the disease. In this review, we would like to highlight the most recent findings in the field of nutrition for the treatment of CD. Our analysis will cover a wide range of topics, from the well-established diets to the new nutritional theories, along with the recent progress in emerging research fields, such as nutrigenomics.

## 1. Introduction

Nutrition has always had a marginal role in the management of Crohn’s disease (CD). A lack of dietary guidelines should be ascribed to the restricted time of clinical visits, to the scarcity of scientific data concerning the effect of alimentation on CD, and to the limited knowledge of nutrition [1]. However, in the past few years there has been a growing interest in nutrition as a critical factor for CD treatment. Awareness of the effects that the environment could exert on the disease pathogenesis, together with a better understanding of the microbiome and its functional role, have fueled scientific research on the impact that diet could have on gut health. This narrative review addresses the most recent dietary recommendations about the nutritional treatment of CD, considering also the latest contributions of emerging fields such as nutrigenomics, with the aim of informing gastroenterologists and improving the patients’ overall health condition.

## 2. Materials and Methods

The purpose of this narrative review is to describe the most recent findings in the nutritional treatment of CD. Three authors, L.L., G.C., and F.C., performed a comprehensive literature search on the electronic databases PubMed, EMBASE, MEDLINE, and Science Direct. Studies were assessed independently, following the narrative review checklist by the Academy of Nutrition and Dietetics. We considered the following terms for the literature search: “Crohn’s Disease”, “Nutritional treatment”, “Nutrition”, “Diet therapy”, “Nutritional strategies”, “Nutraceutical”, “Nutrigenomics”. The logical operators “AND” and “OR” were applied to combine different sets of results. The reference list of the collected papers was also considered to find any relevant articles. Included articles fulfilled the following criteria: (1) described nutritional approaches for the treatment of Crohn’s disease; (2) published in the last ten years (January 2010–December 2020); (3) written in English; (4) full text available. Articles not addressing the inclusion criteria or not pertinent were excluded.

## 3. Crohn’s Disease: Cause and Pathophysiology

CD is a chronic idiopathic inflammatory bowel disease that causes skip lesions and transmural inflammation from mouth to anus [2]. CD prevalence is increasing worldwide in adults and children, and its onset is often characterized by common presenting symptoms such as diarrhea, abdominal pain, rectal bleeding, fever, weight loss, and fatigue [3]. Endoscopy and cross-sectional imaging are the gold standard approaches used to establish a diagnosis and the extent of CD [3]. In addition, laboratory findings, such as thrombocytosis, C-reactive protein (CRP), and some stool markers, i.e., fecal calprotectin, are useful screening tests to assess the disease [4].

CD treatment is focused on the control of the inflammation and the induction of clinical remission; depending on the disease severity, it includes pharmacologic therapy based on 5-5-aminosalicylic acid/mesalazine up to corticosteroids to relieve symptoms. Patients at higher risk are treated with biologics, with or without concomitant immunomodulators, to induce and maintain remission [3]. In some patients, surgery is mandatory; however, in most cases, surgery is not completely effective, and medical therapy is needed to manage CD recurrence.

Although the exact etiology is still unknown, it is believed that the interplay between different factors such as genetic predisposition, environmental factors, microbiota dysbiosis, and defects affecting the innate immune system and the gut barrier functions can trigger the onset of CD [4]. Genetic heritability can be identified in up to 12% of CD patients, with a risk of disease onset that is higher in some populations, such as Ashkenazi Jews vs. others, and Afro-Americans and Asians [4]. Of the 200 alleles associated with IBD, 37 were found to be CD-specific. The NOD2, ATG16L1, LRRK2, IRGM, Il23R, HLA, STAT3, JAK2 genes, and Th17 pathways have been associated with host–microbe interactions, Th17-cell function, and MUC2-related altered mucus layer [5,6]. These findings highlight the importance of bacteria in disease development. However, genetic variations were shown to be causative only in a minority of cases, thus implying that other players, e.g., epigenetic factors, can contribute to CD.

Other relevant mechanisms involve a number of environmental factors. Indeed, a Western life style (with a diet rich in saturated fat and poor in fibers), antibiotic exposure during the childhood, and smoking addiction have all been implicated in the onset of the disease. Dysbiosis has been thought to play a crucial role in CD pathogenesis. Bacteroides, *Firmicutes*, and *Faecalibacterium prausnitzii* species were all demonstrated to be reduced in CD patients, whereas Gammaproteobacteria and Actinobacteria were increased [7,8,9]. Moreover, one-third of CD patients show an increase of mucosa-associated *Escherichia coli*. This adherent-invasive strain is able to cross the mucosal barrier and replicate within macrophages, causing the production of tumor necrosis factor-α (TNF-α). Although microbiota manipulation is a growing research field, thus far the evidence is still too limited to propose probiotics and prebiotics as a treatment option for CD. Moreover, defects of the gut barrier may exert a contributory role in CD pathogenesis. Emulsifiers that are present in the Western diet, together with intestinal tight junction leakage, MUC2 mutations, and autophagy-related ATG16L1 and IRGM genes, can promote intestinal barrier injury and inflammation, thereby eliciting the onset of CD [4,5].

Immune system dysregulation is also involved in CD. Innate lymphoid cells (ILCs) contribute to the maintenance of intestinal barrier integrity. When an offending chemical agent/germ is introduced with the diet, it evokes the synthesis and release of cytokines, such as TNF-α, interleukin 17, interleukin 22, and interferon-γ, by a number of immune/inflammatory cells. Among ILCs, ILC1 and ILC3 are directly involved with CD pathogenesis. Intra-epithelial and lamina propria ILC1 were found to be abundant in the ileum of CD patients [10]. Increased gene expression of key ILC3 cytokines (IL17A and IL22), transcription factors (RORC and AHR), and cytokine receptors (IL23R) has been shown in the inflamed areas of patients with Crohn’s colitis [11].

CD manifestations occur according to three phenotypes: inflammatory, stenosing, and penetrating forms. Regardless of the phenotype considered, in a third of patients there is a perianal involvement, while extra-intestinal symptoms involving the eyes, hematologic system, joints, and skin may also occur [3]. In the majority of CD patients, the most affected GI segments are the terminal ileum and colon, whereas the least involved is the mouth (5% of cases) [12]. At this level, “cobblestone” (fissuring and serpiginous) ulcerations, along with gum swelling and infiltration, are signs detectable in patients with active disease [12]. The oral involvement, together with other symptoms, prevent patients from following a correct diet, resulting in inadequate nutritional support.

## 4. Crohn’s Disease and Nutritional Deficiencies

Malnutrition is often the natural consequence of IBD, detectable in about 65–75% of patients with CD and 18–62% of patients with ulcerative colitis (UC) [13]. IBD-related malnutrition can be ascribed to various mechanisms: (1) reduced intestinal absorption; (2) gut microbiota changes, i.e., intestinal dysbiosis (a typical example of these abnormalities is given by small intestine bacterial overgrowth); and (3) symptoms such as the loss of appetite, nausea, and vomiting. Nutritional deficiencies including folate, vitamin A and D are common among CD patients [3,14]. Moreover, subjects who undergo extensive bowel resection have an increased risk of vitamin B12 malabsorption [15]. Some key nutritional elements, including magnesium, zinc, and iron, can be deficient [15,16]. Compared to UC, patients with CD show lower levels of hemoglobin [14]. Notably, nutritional concerns are particularly important in adolescents with inactive CD, who show an increased energy consumption that is not addressed by an adequate caloric intake [17]. In children, although not associated with increased resting energy expenditure, CD flare-ups compromise the nutritional status by diverting energy from growth to disease activity [17]. For this reason, in CD adolescents it is recommended to address nutritional therapy towards an increased caloric intake to improve both growth and development potential.

In this narrative review, we will describe the most commonly used dietary strategies for the treatment of CD and the latest findings in nutrigenomics, in order to inform and provide practical guidance to physicians and gastroenterologists.

## 5. Diets for the Treatment of Crohn’s Disease

### 5.1. Liquid Diets: Enteral Nutrition and Parenteral Nutrition as Artificial Diets for the Preoperative Nutritional Optimization in CD

Despite significant advances in medical care, stricturing or penetrating complications are very common in about 70% of CD patients, requiring elective surgery within the first 20 years of diagnosis [18,19,20]. Post-operative complications are common in patients undergoing intestinal resection, with a risk rate of 30% in the pre-biologic era [21,22], and lowered to 21% in recent times [23]. Poor nutritional status and a reduction of more than 10% in the body weight during the 6 months preceding surgery were generally associated with the worst post-operative outcomes [21,24,25,26,27]. Nutritional support could attenuate the inflammatory process of the gut, leading to bowel rest and improving postoperative prognosis. In CD patients, enteral nutrition (EN) and parenteral nutrition (PN) are recommended by the European Crohn’s and Colitis Organisation (ECCO) and by the guidelines of the European Society of Clinical Nutrition and Metabolism (ESPEN) for malnourished patients undergoing major gastrointestinal surgery and/or as a minor supportive therapy in addition to an oral diet [28,29,30].

The two artificial nutrition methods will be discussed and compared in the next paragraphs.

### 5.2. Enteral Nutrition

Enteral nutrition (EN) is a liquid dietary regimen, which excludes solid food, providing the full amount of necessary calories. The use of this type of diet is particularly recommended during relapse of the disease, when it is applied for 6–8 weeks to induce remission. EN is administered orally, as a drink, powder, dessert-like snack, or via a feeding tube, with similar efficacy [31]. To date, EN can be delivered in three formulations, depending the on protein and fat content: elemental, semi-elemental, and polymeric. Elemental formulas contain low-fat nutrients such as amino acids, mono- or oligo-saccharides, and medium-chain triglycerides that are easily absorbable. Semi-elemental formulas consist of peptides of different chain length, simple sugars, glucose polymers or starch, and medium-chain triglycerides. Finally, polymeric formulas contain whole proteins, complex carbohydrates, and long-chain triglycerides [32].

EN has also been recommended as a maintenance diet during the remission phases of CD, combined with the usual diet [33]. A maintenance enteral diet (MEN) has been shown to increase the positive effects of biological therapies (e.g., Infliximab), thus preventing the relapse of the disease after surgical-induced remission [34,35,36,37,38]. EN can also be administered as the only nutrition treatment, i.e., exclusive enteral nutrition (EEN). Besides the primary function, EEN provides other beneficial effects, such as improving the nutritional status and bone metabolism/turnover in children [34,39,40]. Indeed, EEN is the main therapy for mild-to-moderate CD in children and adolescents, as this regimen promotes, beside the remission of the illness in 80–85% of the cases [41,42,43], a reduced use of steroids, which are known to impair growth. However, despite the promising results of EEN over steroids in the treatment of pediatric CD [41,42,44], similar results have not been obtained in adult patients, since corticosteroids still show better remission rates when compared to EEN [37,45,46]. EEN is also suggested for the remission of complicated CD, improving the inflammatory strictures or entero-cutaneous fistulas [1,47,48].

EEN can affect the gut microbiota, i.e., the myriad of bacteria, archaea, eukarya, fungi, and viruses resident in the gut lumen [49]. It is currently accepted that intestinal dysbiosis (that is the altered richness and diversity of the microbiota) is one of the main trigger factors contributing to CD. Bacterial species that are altered in dysbiosis include *Bacteroidetes* and *Firmicutes*, together with deficiency of *Faecalibacterium prausnitzii*, a strain expressing a 15k Da protein with anti-inflammatory properties [8,9,50]. Furthermore, the prevalence of *Enterobacteriaceae* (*Salmonella*, *E. Coli*, and *Campylobacter* spp.) has been associated to IBD, although it is still unclear whether the overgrowth of these species is the cause or a consequence of the disease [41,51].

Studies have shown that EEN reduces bacterial richness in children via the reduction of Bacteroidetes species [50,52,53] and the increase in *Firmicutes* phylum and T-regulatory cells of the intestinal *lamina propria*. EEN also promotes a reduction in the fecal calprotectin levels, a marker of gut inflammation [50,54,55]; however, this effect is rapidly lost after food re-introduction [50,53,55]. Moreover, EEN reduces the operational taxonomic units (OTU) [50,56], which is an index of bacterial diversity in adults.

Together with the reduction of possibly detrimental bacterial species, EEN evokes the reduction of *F. prausnitzii* spp. and of fecal butyrate production [52] 30 days after treatment, thus providing a rationale for supplementing butyrate to EEN [50]. Limitations of EEN are the poor palatability and the difficulty experienced by patients in following a liquid diet for a long period of time. All these factors hamper the patients’ compliance to the treatment [32]. In the attempt to make the EEN more palatable, a partial EEN has been proposed, where EEN has been combined with *ad libitum* solid food; however, this approach did not guarantee a similar rate of remission as total EEN [1,57]. Zoli et al. compared an elemental diet administered orally with a high-dose steroid therapy. The randomized study investigated 22 adult patients affected by moderately active CD. After two weeks of treatment, patients treated with the oral elemental diet achieved the same clinical and laboratory remission as patients treated with corticosteroids, thus proving that an oral elemental diet could be as effective as steroids in inducing CD remission in adults [58].

### 5.3. Parenteral Nutrition

Parenteral nutrition (PN), along with its exclusive form total parenteral nutrition (TPN), provides nutrients (macronutrients, micronutrients, and electrolytes) through a central venous catheter [59,60]. In agreement with the ECCO current practice position 2.3: “PN in patients with CD can optimize nutritional status prior to surgery as a supplement to EN, or as an alternative if the use of EN is not possible or is contraindicated” [61]. PN is commonly recommended for malnourished patients who are experiencing an acute inflammatory phase, with the aim of achieving bowel rest. Additionally, PN is recommended when postoperative complications affect gastrointestinal function, and it is difficult to feed patients with oral/EN for at least 7 days [62]. Other features making PN feasible include bowel obstruction or sub-occlusion, high-output fistulae, bowel ischemia, severe hemorrhage, anastomotic leak, or active disease causing gut dysfunction [30,61]. A systematic review by Comeche et al. [60] showed that PN ameliorates erythrocyte sedimentation rate [63,64], cholesterol [60,65], total phospholipids [65], and serum albumin [65,66,67], without producing the clinical symptoms of hypoglycemia [68]. Moreover, some studies found a significant reduction in the CD activity index (CDAI) after PN administration [64,69,70], although these results were not confirmed by Ockenga et al. [71]. However, despite these improvements and the concomitant use of immunosuppressive drugs, antibiotics, and fecal microbial transplantation, relapses of malabsorption remain frequent in CD patients [72].

### 5.4. Enteral Nutrition and Parenteral Nutrition for Safer Elective Surgery and Reduced Post-Operative Complications in Adults with CD

Heerasing et al. [18] conducted a retrospective case-control study to determine whether EEN, administered for at least 2 weeks prior to surgery, could improve post-operative complications in adult CD patients requiring surgery for stricturing or penetrating complications. Their findings showed that EEN reduced the need for surgery in 25% (13/51) of patients, shortening the length of stay in a surgical unit and dampening systemic inflammation (overall, serum CRP values dropped from 36 mg/L to 8 mg/L in the EEN treated patients). Moreover, patients who were referred to surgery displayed a nine-fold increase in the incidence of post-operative abscesses and/or anastomotic leak compared to those who were pre-treated with EEN. Similar results were obtained by Yamamoto et al. [73] in 24 CD patients receiving EN for 2–4 weeks before surgery compared to 24 untreated (control) patients. In the EN treated arm, the median serum albumin levels increased, while CRP significantly decreased. Furthermore, the incidence of septic complications (anastomotic leak, intra-abdominal abscesses, entero-cutaneous fistulas, or wound infection) was significantly reduced in patients who received EN pre-operatively (4% vs. 25%, *p* = 0.04). A retrospective study on 123 CD patients by Li et al. found similar results [74]. Fifty-five patients (44.7% of the total) were fed with EEN for 3 months prior to surgery, showing significantly higher serum albumin levels and lower CRP at operation, and showing a lower risk of intra-abdominal septic complications (IASCs) (3.6% vs. 17.6%, *p* < 0.05). Three months after surgery, IASCs occurred in 14 patients (11.4% of the total), nine with anastomotic leakage (of these only one received pre-operative EEN) and five with intra-abdominal abscesses (only one had pre-operative EEN). However, despite the lower number of post-operative complications observed in patients fed with EEN, two years after surgery the cumulative risk of IASCs was similar in the two groups (*p* = 0.109).

A retrospective study analyzed the effect of TPN over 30-day infectious complications in 144 malnourished CD patients who underwent major abdominal surgery; 55 patients had pre-operative TPN vs. 89 untreated (control) patients [21]. The study concluded that receiving TPN for ≥60 days before surgery had a significantly lower risk of post-operative non-infectious complications compared to the controls (*p* = 0.03).

Jacobson [65] compared the effect of pre-operative TPN administered for 18–90 days to 15 patients undergoing bowel resection and primary anastomosis with 105 matched controls. All the patients of the TPN group displayed clinical remission of CD (general well-being and improvement of abdominal pain, fever, and diarrhea). Moreover, postoperative complications occurred only in the control group (29 patients out of 105, a statistically significant result). Thus, TPN is recommended for patients with moderate to severe CD for at least a period of 18 days before major intestinal surgery.

### 5.5. Enteral vs. Parenteral Nutrition

An ESPEN panel of experts [30] conducted a systematic review exploring the prognosis of surgical patients treated with EN vs. PN. The authors reviewed twenty randomized studies recruiting patients with abdominal surgery, including patients after liver transplantation and trauma [30]. Six of the fifteen studies comparing PN directly with EN indicated the latter as the preferred artificial nutrition, due to the lower incidence of infectious complications, shorter length of stay, and best cost–benefit. Eight studies found no significant difference between EN and PN, and for this reason, they suggested using EN because of limited costs. A meta-analysis on 27 studies of TPN conducted by Heyland et al. [75] found a lower complication rate in surgical patients receiving TPN, compared to no TPN or standard care (usual oral diet with intravenous dextrose), especially in those with malnutrition. The high heterogeneity of the analyzed studies hampered the decision as to whether EN was better than TPN or vice versa. Another meta-analysis by Braunschweig et al. [76], comparing EN to PN in 27 studies with a total of 1828 patients, showed that the risk of infection is lower with oral/enteral nutrition, whereas in a subcategory of malnourished patients, infection and mortality rates were significantly reduced for those treated with PN. Peter et al. [77] found lower infection rates and a shortened length of hospitalization for EN fed patients. ESPEN 2017 guidelines state: “if oral feeding is not sufficient then tube feeding should be considered as supportive therapy. Enteral feeding using formulae or liquids should always take preference over parenteral feeding, unless it is completely contraindicated” [29]. However, although EN should always be preferred to PN, combined EN and PN may be considered in patients needing nutritional support and those in whom >60% of energy cannot be provided solely by EN (because the integrity of the gastrointestinal tract is compromised or due to intestinal dysfunction). Thus, EN often represents the main treatment option, alone or in association with PN [78].

### 5.6. Specific Carbohydrate Diet (SCD)

The SCD was developed in the 1920s for the treatment of celiac disease and then adopted by the gastroenterologist Dr. Sidney Haas in 1951 for the treatment of IBD [79]. SCD allows the consumption of monosaccharides, excluding disaccharides and most of the polysaccharides. In SCD, permitted foods include meat, eggs, oil, vegetables rich in amylose, dairies with low lactose content, e.g., dry-curd cottage cheese or home-made 24-h fermented yoghurt, nuts and fruits (all types). “Forbidden” SCD foods are sucrose, maltose, isomaltose, lactose, potatoes, okra, corn, fluid milk, soy, cheeses with a high amount of lactose (e.g., fresh cheese), food additives, and preservatives. Moreover, Gottshall suggested SCD for at least one year after symptom cessation; for this reason, it could be difficult to strictly adhere to this diet for various (e.g., working or social) reasons. Another study showed that SCD improves symptoms and patient quality of life, and in some cases maintained the remission without the need for medications [80]. In children, SCD also promotes the mucosa healing assessed with Lewis score [1,81] and induces the normalization of inflammatory markers, such as CRP, fecal calprotectin, and serum albumin [82,83,84]. A research project, promoted by the Patient-Centered Outcomes Research Institute (PCORI) and not yet concluded, aims to compare the SCD to the Mediterranean diet in terms of symptom remission in CD. The results will help to determine whether the Mediterranean diet, recommended for the treatment of many different conditions, should be considered in the management of CD patients [85].

### 5.7. Low FODMAP Diet

The acronym FODMAP stands for fermentable, oligosaccharides, disaccharides, monosaccharides, and polyols. The low FODMAP diet was initially created for IBS patients and then was also proposed for the treatment of IBD conditions. This diet is based on the exclusion of short-chain carbohydrates, which are poorly absorbed and highly fermented by intestinal bacteria, thereby promoting diarrhea, bloating, distention, and abdominal pain [80]. Patients on a low FODMAP diet should limit honey and some fruits, such as apples, dates, watermelon (source of fructose), onions and garlic (source of fructans), beans, lentils, and legumes (source of galactans), while there are no restrictions concerning the use of sucrose. Although this type of diet is associated with an improvement of gastrointestinal symptoms [1,86], there is no evidence for an improvement of calprotectin levels or of the reduction of the luminal inflammation [87]. The low FODMAP diet is advisable in patients with quiescent IBD [88] exhibiting IBS symptoms detectable in up to 57% of CD patients [89,90]. The downside of this diet is the reduced intake of inulin, fructo-oligosaccharides, and fructose, which are known prebiotics [79]; moreover, the FODMAP diet reduces the *Bifidobacterium* population [91], thus enhancing dysbiosis [92].

### 5.8. Semi-Vegetarian Diet (SVD)

The SVD, also referred to as “flexitarian”, describes a primarily vegetarian dietary regimen, which is “flexible” meaning that it strongly limits meat and fish, without eliminating them. The SVD is based on vegetable, fruits, cereals, eggs, yoghurt, and milk, while excluding all processed and refined foods [79]. Chiba et al. [93] carried out a 2-year clinical study administering SVD to CD patients in medically or surgically induced clinical remission. This was maintained in 15 of 16 patients on SVD (94%) vs. two of six (33%) patients who followed a free diet. Maintenance of remission rates with SVD was 100% at one year and 92% at two years, suggesting that SVD is effective in preventing CD relapses. In a case report by Sandefur et al. [94], a patient affected by CD for three years and who had been on infliximab for two years experienced a complete resolution of symptoms after 40 days of vegetarian diet and processed food avoidance for religious purposes. Thus, the patient decided to continue on a vegetarian diet, with rare periods of poor compliance (notably all accompanied by symptom relapse). Six months after switching to a full plant-based diet, complete mucosal healing with no visible evidence of CD was reported at follow-up ileo-colonoscopy. The same group [95] investigated the remission maintenance rate in CD patients treated with a lacto-ovo-vegetarian diet in which additional servings of fish once a week and meat once every two weeks were included. The proposed diet was particularly rich in fibers (soluble 6.8 ± 0.7 g vs. 23.3 ± 1.6 g insoluble dietary fiber), exceeding the recommended daily dose for the Japanese population (17 g/day for women and 20 g/day for men). Maintenance of remission in patients on the lacto-ovo-vegetarian diet was 92% at two years, and without therapy with biologic drugs, suggesting that a high-fiber content diet can be indicated in the management of a subset of patients with CD.

### 5.9. Other Diets

In recent years, some new dietary approaches have emerged. However, the lack of clinical trials and scientific data suggests a cautious approach to their uncontrolled use. Here we report the most popular ones. The low fat/fiber limited exclusion (LOFFLEX) diet, which follows the elemental (liquid) formula, is basically a way of reintroducing foods avoided for their potential to trigger CD. The LOFFLEX diet helps apply the exclusion of nutrients in a well-structured protocol. Paleolithic, maker’s, and vegan diets are all regimens applied with some presumed efficacy and generally promoted by the media or the lay press, although devoid of actual scientific evidence.

Among elimination diets, the gluten-free diet (GFD) has undoubtedly sparked interest. Indeed, a genetic predisposition for celiac disease may evoke the onset of IBD, although a causative relationship between celiac disease and IBD has never been fully established [96,97]. To date, despite some experimental data on animal models showing that gluten triggers intestinal inflammation and increases epithelial barrier permeability, no clinical trials have clearly indicated that a GFD has effects on CD. An internet-based survey of 1647 patients with IBD conducted in the United States reported that 65.6% of patients on a GFD for the first time experienced an improvement of symptoms associated with the disease (nausea, bloating, diarrhea, abdominal pain, fatigue) [98]. Conversely, a Swiss study did not show any significant clinical improvement following a GFD [99]; notably, those who adhered to a GFD reported worsening of the psychological condition. Overall, the scarcity of data and the significant dissimilarities among studies prevent a clear answer as to whether a GFD could have an effect on IBD and in particular on CD. More investigations are eagerly awaited on GFD in IBD patients.

**Figure 1:**
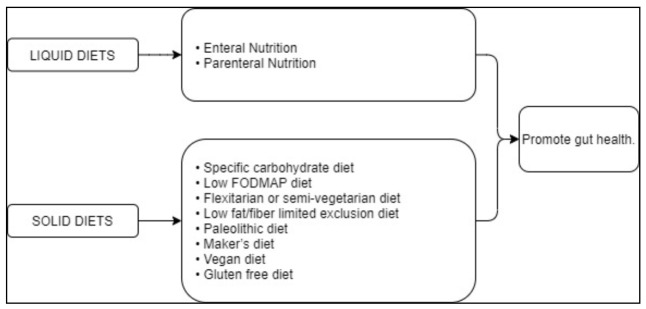
Synoptic view summarizing the different dietary regimens for the treatment of CD.

The suggested diets for the treatment of CD are summarized in Table 1 and schematically illustrated in Figure 1.

## 6. Probiotics, Prebiotics, and Symbiotics

Probiotics are bacteria able to reach the small intestine and the colon alive, providing beneficial effects to the gut microbiota of the host. Probiotics exert an antimicrobial effect, and promote intestinal epithelial barrier integrity and the improvement of the host immune response [100]. For these reasons, they are increasingly recommended in addition to dietary modifications during illness (i.e., diarrhea), antibiotic use, or other conditions evoking gut dysbiosis. Probiotics could also be recommended to healthy people, to maintain physiological functions and/or avoid the onset of pathological conditions [101]. Today, probiotics are gaining interest and research has focused on their effects in IBD. Although the use of probiotics, alone [102] or in combination with 5-ASA, seems to be promising [103,104] for the treatment of UC, their efficacy seems to be uncertain for the treatment of CD [105]. In particular, the VSL#3 formula, containing a mix of eight bacterial species, has been investigated both in UC and CD patients. Although the VSL#3 formula had the same effect of mesalaxine treatment when administered to UC patients [106], similar results were not achieved in CD. Fedorak et al. [107] found that the VSL#3 formula reduced the level of inflammatory cytokines in the mucosa and delayed the disease recurrence in CD patients administrated with it for the entire 365 days. However, there was no statistical difference in endoscopic recurrence rates registered at day 90 between patients treated with VSL#3 formula and patients treated with placebo [107]. The anti-inflammatory properties of this strain suggest its use as a promising probiotic adjuvant to CD therapy.

Results on the use of probiotics in children are contradictory. In children affected by mild to moderate CD, Lactobacillus GG proved to be effective in increasing the intestinal barrier function [108]. Conversely, another study showed that Lactobacillus GG failed to delay CD recurrences in children with CD [109].

Yilmaz et al. investigated the effects of Kefir drink, a fermented dairy product, on patients affected by CD and UC [110]. Kefir contains a mixture of probiotics that degrade the lactose contained in milk, and therefore it can be consumed by patients with lactose intolerance. The researchers isolated and identified six different strains of *Lactobacillus* in Kefir (*L. pentosus*, *L. brevis*, *L. plantarum*, *L. fermentum*, *L. kefiri*, and *L. lindneri*). Patients received 400 mL/day of Kefir, twice a day, for four weeks. After one month, CD patients reported a statistically significant improvement of abdominal pain and bloating along with a higher feeling of wellbeing compared to UC patients. Moreover, Kefir treatment in CD patients elicited a significant reduction of inflammatory parameters, such as erythrocyte sedimentation rate and CRP, associated with hemoglobin level increase. Furthermore, Kefir could have potential immunomodulatory effects, due to the probiotic ability to restore intestinal permeability.

Prebiotics, defined as dietary compounds able to nourish the commensal bacteria, were found to not improve the CD activity index nor the endoscopic score or the immunohistochemistry. A study by Benjamin et al. on 103 CD patients randomized to receive either fructooligosaccharides (FOS) or placebo found that, although FOS enhanced Bifidobacteria and *F. prausnitzii* in healthy subjects, they do not have the same effects in patients with active CD [111].

A study by Halmos et al. [87] investigated the prebiotic effect of a low FODMAP diet vs. CD patients randomized in a cross-over design to receive either a low FODMAP or typical Australian diet for 21 days. Feces were collected at the end of each diet and analyzed for calprotectin, pH, SCFA, and bacterial abundance. Gastrointestinal symptoms were reported daily. Apart from an improvement of gastrointestinal symptoms (i.e., abdominal pain, bloating, and passage of wind), there was no difference in stool pH and total or specific fecal SCFA in patients fed with low FODMAP compared to those following the Australian diet. Gut microbiota abundance was unchanged between the two groups; however, specific bacteria changed in those who were in the FODMAP diet arm. Indeed, *A. muciniphila*, a bacterial population associated with beneficial effects on CD, diminished, and *R. torques*, which is abundant in patients with IBD, increased. *Lactobacilli* and *Bifidobacteria* spp., typical markers of the prebiotic activity, did not change between the two groups. Thus, a low FODMAP diet in CD patients could relieve the typical functional symptoms, although its use should be approached carefully, as a long-term restriction of FODMAP intake reduces the prebiotic effect, leading to potentially dangerous changes of gut microbiota. Finally, because of the limited number of investigated patients, the results of Halmos’ study should be carefully considered.

The combined use of probiotics and prebiotics, referred to as symbiotics, has led to interesting results. Steed et al. used a symbiotic formula containing Bifidobacterium longum and Synergy1 randomly administered to 35 CD patients vs. a placebo [112]. The results showed a significant improvement of CDAI (tested at 3 and 6 months) and histological scores. The symbiotic did not exert a significant effect on the mucosal IL-18, INF-γ, and IL-1β, whereas it reduced TNF-α expression (only at 3 months of treatment) and promoted mucosal Bifidobacteria proliferation.

In conclusion, probiotics, prebiotics, and symbiotics represent a possible dietary integration for CD patients, but the existing scientific literature reports a very mild influence on CD patients’ clinical and laboratory improvement, thus requiring more studies to define their actual efficacy.

**Figure 2:**
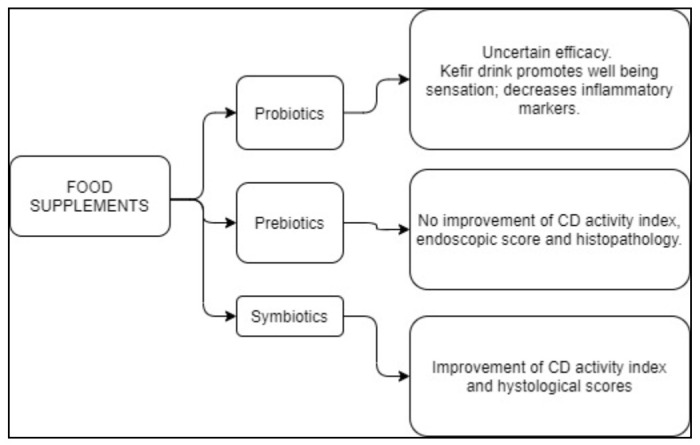
Suggested food supplements for the treatment of CD.

Dietary supplements are summarized in Table 2 and schematically illustrated in Figure 2.

## 7. Nutrigenomics

The term “nutrigenomics” refers to an area of research that investigates the effects that foods may have on gene expression. According to the concept that “no one-size fits all”, the goal of nutrigenomics is to create a “tailored” nutrition. This personalized approach will make possible the creation of customized diets according to each individual’s genotype, with the help of biochemistry, physiology, epigenetic modifications, microbiome, nutrition, and the “omic” disciplines: genomics, proteomics, metabolomics, and transcriptomics [113]. Thus, genetics can indicate the gene affected, and a specific food component could be recommended (for example, long-chain n-3 PUFA or fish oil in case the interleukin gene is mutated, or prebiotics/probiotics, should the *NOD2* or *ATG16L1* gene be affected) [114]. The effect of foods, alone or in combination with other therapeutic strategies, could be determined using transcriptomics [114,115], while the long-term effects of diet could be investigated using proteomic and/or metabolomic approaches [116,117].

CD was one of the first diseases investigated by genome-wide association studies (GWAS). More than 200 genes have been related to IBD susceptibility, some of which were also involved in the immune-mediated disorders, ankylosing spondylitis and psoriasis, whereas others modulate the host–microbiota interactions [118]. Research on food components unveiled their pivotal contribution in gene expression modulation, metabolic pathway activation, transcription factors, and epigenetic modification. Micronutrients, such as vitamin D, vitamin E, calcium, folic acid, retinol, and nicotinic acid, have been associated with reduced DNA damage and are highly recommended in an appropriate dose for the treatment of CD patients, as they play a role in inflammation and immune response [119,120]. Indeed, it has been demonstrated that polymorphisms affecting the human receptor of vitamin D promote susceptibility to IBD [121,122,123]. Among food components, the green tea polyphenol, epigallocatechin gallate (EGCG), further to its antioxidant properties, is able to influence many functions involved in CD pathogenesis, such as methylation, transduction, transcription factors, mitochondrial function, and autophagy, and limits the activation of the signal transducer and activator of transcription 3 (STAT3) pathway involved in CD development [124]. Other nutrients, such as fibers, may modulate gene signaling. In CD, dietary fibers are not recommended during the exacerbation of the disease. Thus, the limited intake of fiber reduces the production of SCFAs by dietary fiber fermenting bacteria [125], down-regulating free fatty acid receptor 2 (FFAR2) gene expression. *FFAR2* is involved in the maintenance of healthy gut microbiota, and mutations affecting this gene could aggravate the tolerance to fiber in CD subjects [126].

However, another study showed that cruciferous (cabbage, broccoli), normally considered a beneficial food for their anti-neoplastic properties, are detrimental for some CD patients. Laing et al. demonstrated that a SNPs on the major histocompatibility complex is responsible for the adverse effects that cruciferous vegetables could exert on CD patients [14]. As this mutation is common in CD patients, it explains why cruciferous vegetables are generally considered beneficial for most people, but not for CD patients.

In addition, CD subjects carrying a single nucleotide polymorphism (L503F, c. 1672 C > T) of the organic cation transporter gene OCTN1 show a higher sensitivity to mushroom compared to people carrying the same mutation but not affected by CD [127]. Other dietary components that influence the genome are fructose, artificial sweeteners, infant formula, food emulsifiers, and antibiotics. High consumption of fructose is one of the factors eliciting IBD onset. Indeed, fructose enhances the expression of the thioredoxin-interacting protein (TXNIP) gene, which evokes hepatic inflammation, and contributes to NF-κB regulation [128,129,130]. Artificial sweeteners, infant formula, food emulsifiers, and antibiotics are associated with gut dysbiosis, another leading factor promoting IBD onset [131,132].

Genetic assets may provoke or counteract the onset of CD. An example of a genetic variation exerting a protective effect on CD is represented by the *PPAR-γ* gene involved in fatty acid storage and glucose metabolism regulation, and thus contributing to inflammation processes and cancer cell growth [133]. In addition, in humans *PPAR-γ* gene products orchestrate the antimicrobial immunity response, maintaining the epithelial expression of colonic beta-defensin DEFB1, which is reduced in CD. Studies on *PPAR-γ* found that the Pro12Ala variant was seen to exert a protective role against CD in a European Caucasian population [134,135]. Thus, subjects carrying the variant have a reduced risk of developing the illness with respect to those who do not.

Carrying specific genetic traits could aggravate the CD condition. For example, absorption of beta-carotene, a precursor of vitamin A, is regulated by the 15,15′-monooxygenase gene (BCMO1) [136]. The enzyme encoded by BCMO1 cleaves beta-carotene into two retinal molecules. Leung et al. discovered that people carrying one of the two gene polymorphisms are not able to convert beta-carotene to retinol [137]. This genetic variation is common in the general population (e.g., 45% of people in the study carried one of the two polymorphisms). Thus, CD subjects bearing such a genetic trait may display a limited production of vitamin A, leading to the aggravation of the disease, as the appropriate production of vitamin A is necessary for the correct regulation of the adaptive immune system and the innate immune defense response [138].

Epigenetic alterations play a primary role in CD orchestration. A recent study shed light on the cross-talk between the miRNA and epigenetic mechanisms implicated in CD development, finding 26 miRNAs highly expressed in CD patients and modulating epigenetic modifications putatively involved with CD progression [139]. In particular, miR-21, which controls the innate and adaptative immune system and hypomethylation of the miR-21 locus, was found to correlate with increased primary miR-21 expression in leucocytes and in inflamed intestinal mucosa [140]. Besides miR-21, other microRNAs (miR-122, miR-29, miR-192, miR-146a) may play a role in CD development; in particular, high levels of circulating miR-595 and miR-1246 are associated with a more aggressive form of CD [141].

The Western diet is characterized by a high intake of saturated fatty acids and low consumption of polyunsaturated fatty (PUFAs) and long-chain PUFAs (LC-PUFAs) [142,143,144,145,146], and for this reason it is considered one of the causes of systemic inflammation. In Western populations, the ratio between omega-6 (one of the factors promoting inflammation) and omega-3 has been estimated to be 10:1–20:1, instead of the optimal estimated ratio, which is 4:1 [147], a condition that is mainly due to the increasing abuse of vegetable oils (e.g., soy, safflower, corn, and sunflower) [142,148,149]. However, this ratio also seems to be influenced by personal genotype. Indeed, mutations or variants in fatty acid desaturase genes (FADS1, FADS2), the peroxisome proliferator-activated receptor genes (PPARA, PPARG), the X-ray repair cross-complementing protein 1 gene (XRCC1), and stearoyl-CoA desaturase gene (SCD1) influence the serum levels of LC-PUFA-omega-3 and omega-6 fatty acids, affecting metabolic pathways, inflammation, and cancer risk [138]. Again, carrying mutations for these genes could become a detrimental factor for CD patients who already have a limited dietary intake of fish and fish oil and a low omega-3:omega-6 PUFA ratio.

Gut dysbiosis could be exacerbated in some genotypes: the loss of function of the fucosyltransferase 2 (FUT2) gene, involved in CD susceptibility [137], reduces the microbiota richness and abundance. People carrying this genetic trait are defined as “non-secretors” and show low colonization of *Bifidobacterium bifidum*, *B. adolescentis*, and *B. catenulatum*/*pseudocatenulatum* [150]. As *Bifidobacteria* are key factors in healthy infant microbiota development and protect from pathogens [151], “non-secretors” IBD will have a greater chance of developing gut inflammation. This may be aggravated by some dietary regimens, such as the low-FODMAP diet, that reduces microbiome richness and decreases *Bifidobacteria* [152].

**Figure 3:**
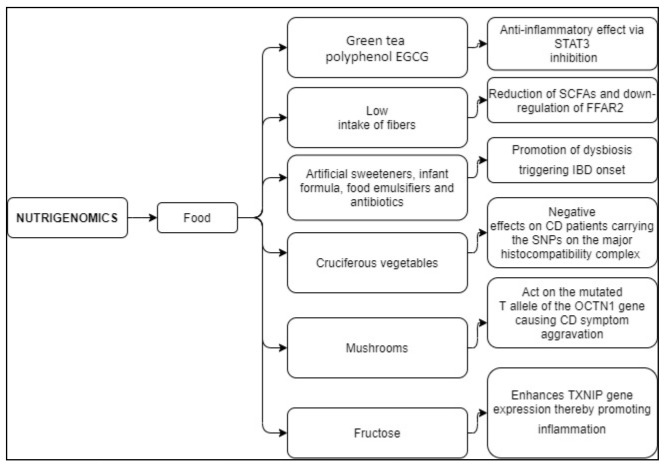
Nutrigenomics: effects of food on the modulation of gene expression and their influence on CD evolution. Abbreviations: EGCG, EpiGalloCatechin Gallate; STAT3, Signal Transducer and Activator of Transcription 3; SCFAs, Short-Chain Fatty Acids; FFAR2, Free Fatty Acid Receptor 2 gene; IBD, Inflammatory Bowel Disease; SNPs, Single-Nucleotide Polymorphism; OCTN1, Sodium-Dependent Organic Cation Transporter gene; TXNIP, Thioredoxin-Interacting Protein; NF-κB, Nuclear Factor Kappa-Light-Chain-Enhancer of Activated B cells.

**Figure 4:**
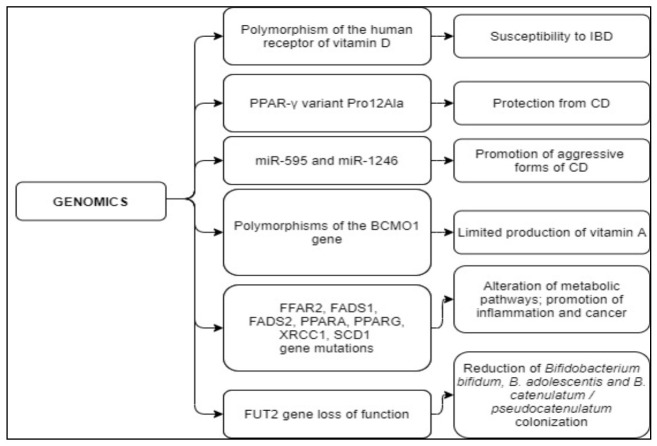
Effects of genetic variation on CD onset and outcomes. Abbreviations: IBD, Inflammatory Bowel Disease; PPAR-γ Peroxisome proliferator-activated receptor gamma; miR-595, MicroRNA 595; miR-1246, MicroRNA 1246; BCMO1, β,β-carotene-15,15′-monooxygenase 1; FFAR2, Free Fatty Acid Receptor 2; FADS1, Fatty Acid Desaturase 1; FADS2, Fatty Acid Desaturase 2; PPARA, Peroxisome Proliferator Activated Receptor Alpha; PPARG, Peroxisome Proliferator-Activated Receptor Gamma; XRCC1, X-ray Repair Cross Complementing 1; SCD1, Stearoyl-CoA Desaturase-1; LC-PUFA-omega-3 and omega-6, Long Chain Polyunsaturated Fatty Acids-omega-3 and omega-6; FUT2, Fucosyltransferase 2.

Among people suffering from IBD, 10–20% of them show dairy product sensitivity [153]. This condition is unrelated to the disease activity status and depends on the presence/absence of the lactase enzyme that catalyzes the milk disaccharide lactose into the two monosaccharides, i.e., galactose and glucose. In most mammals, lactase stops being active after weaning; however, in humans, it can persist into adulthood. This condition, called “lactase persistence”, is present in subjects heterozygous or homozygous for the T allele of DNA variant, rs4988235, located 14 kb upstream from the lactase phlorizin hydrolase (LCT) gene locus, whilst those homozygotes for the C allele of rs4988235 show lactase-non persistence [154]. Nolan et al. demonstrated that Caucasian people in New Zealand have a strong association between lactase persistence genotype and risk of developing CD. Indeed, subjects homozygous for the T allele show a higher risk of developing CD compared to those homozygous for the C allele [155]. Overall, these findings suggest that CD onset could be induced by a variety of factors including the complex relationships underlying the crosstalk between food components and genotype. The main findings on nutrigenomics and genetic modifications have been summarized in Table 3 and Table 4, and schematically summarized in Figure 3 and Figure 4.

## 8. Conclusions

Consistent data indicate that CD is a condition originating from the complex interplay among different factors, i.e., gene abnormalities, altered immune response, and environmental and gut microbiota changes. In this scenario, nutrition, ranging from dietary manipulation to EN/PN, plays an essential role in the treatment of IBD, and in particular of CD. Indeed, it is becoming clear that food components have the ability to modulate metabolic pathways, stimulate gene expression, and modify the microbiota composition. Liquid diets represent the primary therapy in CD treatment, as they reduce inflammation and promote mucosal healing, as well as reducing post-operative complications. Besides the classical dietary approaches, new functional foods are being explored, and new technologies, defined by the suffix “omic”, are being developed to investigate the underlying relationship between food and genes. Although the application of these technologies to CD is still at its very beginning, the idea that every patient is somehow unique prompts *ad hoc* treatments based on specific diets and nutrient intake. Hopefully, in the next few years, a holistic strategy will allow the treatment of CD patients through personalized nutritional approaches.

## Figures and Tables

**Table 1:** Suggested dietary approaches for CD treatment with explanation of mechanisms of action and effects.

Dietary Treatment			Putative Action	Clinical Impact
Enteral nutrition (EN)			Promotes gut health [29]	EN promotes CD remission [33]
Parenteral nutrition (PN)			Promotes gut health [63]	PN favors CD remission. This diet is particularly indicated for malnourished patients during an acute inflammatory phase or post-operative complications affecting gastrointestinal function [61,62]
Specific carbohydrate diet (SCD)			Promotes gut health. “Forbidden” foods are sucrose, maltose, isomaltose, lactose, potatoes, okra, corn, fluid milk, soy, cheeses with a high amount of lactose such as fresh cheese, food additives, and preservatives [79]	SCD improves symptoms and quality of life and, in some cases, maintains remission with no need of medications [79]. In children, SCD promotes mucosal healing [81]. SCD normalizes inflammatory markers, e.g., CRP and fecal calprotectin, and serum albumin [82,83,84]
Low FODMAP diet			Promotes gut health. “Forbidden” foods are fermentables, oligosaccharides, disaccharides, monosaccharides, and polyols [86]	Improved gastrointestinal symptoms [1,86]; no evidence that calpotectin levels or luminal inflammation ameliorate [87]
Flexitarian or semi-vegetarian diet (SVD)			It promotes gut health. Limited amounts of meat and fish are allowed [93]	SVD is effective in preventing CD relapse [93]
Low fat/fiber limited exclusion diet (LOFFLEX)			Elemental formula followed by an exclusion diet in a well-structured protocol [79]	Possible induction of CD remission although its efficacy is not yet fully demonstrated [79]
Paleolithic diet	Maker’s diet	Vegan diet	Elimination diets [79]	Efficacy not demonstrated yet [79]
Gluten free diet			Absence of gluten intake [79]	Contrasting data [79]

Abbreviations: EN, Enteral Nutrition; PN, Parenteral Nutrition; CD, Crohn’s Disease; SCD, Specific Carbohydrate Diet; FODMAP, fermentable, oligosaccharides, disaccharides, monosaccharides and polyols; SVD, Flexitarian or Semi-Vegetarian Diet; LOFFLEX, Low Fat/Fiber Limited Exclusion Diet.

**Table 2:** Summary of the main features related to prebiotics, probiotics, and symbiotics.

Food Supplement	Mechanism of Action	Clinical Impact
Probiotics	Mainly bacteria able to reach the small intestine and the colon alive, providing positive interaction with gut microbiota of the host. Probiotics may exert various beneficial effects, including antimicrobial action, mucosal integrity, and enhancing the host immune response [100]	Uncertain clinical efficacy in CD patients. Kefir drink, a probiotic mix, improves abdominal pain, bloating, and inflammatory markers, along with increasing wellbeing sensation [110]
Prebiotics	Indigestible dietary compounds fueling beneficial bacteria of the gut microbiota	No major improvement of CD activity index, endoscopic score, or histopayhology [87,111]
Symbiotics	Combination of probiotics and prebiotics	A symbiotic containing Bifidobacterium longum and Synergy1 improved CD activity and histological scores [112]

**Table 3:** Food/dietary components affecting gene expression or other factors with related mechanisms and effects.

Food/Dietary Component	Putative Mechanism	Effects
The green tea polyphenol EGCG	Limits the activation of the STAT3 pathway [114,124]	Anti-inflammatory effect [114,124]
Low intake of fibers	Reduced SCFAs production by dietary fiber fermenting bacteria, down-regulating the FFAR2 signaling [125,126]	FFAR2 mutations worsen fiber tolerance in CD patients [126]
Artificial sweeteners, infant formula, food emulsifiers, and antibiotics	Promote dysbiosis [131,132]	Increased risk of IBD onset [131,132]
Cruciferous vegetables	Antioxidant effects [14]	Detrimental effects on CD patients carrying the SNPs on the major histocompatibility complex [14]
Mushrooms	Act on the mutated T allele of the OCTN1 (c. 1672 C > T) gene [127]	People suffering from CD and carrying the genetic mutation show mushroom sensitivity [127]
Fructose	Enhances TXNIP gene expression [128,129,130]	Promotes inflammation in endothelial cells, eliciting hepatic inflammation, and contributes to NF-κB regulation [128,129,130]

Abbreviations: EGCG, EpiGalloCatechin Gallate; STAT3, Signal Transducer and Activator of Transcription 3; SCFAs, Short-Chain Fatty Acids; FFAR2, Free Fatty Acid Receptor 2 gene; IBD, Inflammatory Bowel Disease; SNPs, Single-Nucleotide Polymorphism; OCTN1, Sodium-Dependent Organic Cation Transporter gene; TXNIP, Thioredoxin-Interacting Protein; NF-κB, Nuclear Factor Kappa-Light-Chain-Enhancer of Activated B cells.

**Table 4:** Genetic abnormalities involved in the onset and/or outcomes of CD.

Genetic Abnormality	Mechanism of Action	Effects
Polymorphisms of the human receptor of vitamin D	The vitamin D receptor form is different from the classical one [121,122,123]	Polymorphism increasing susceptibility to IBD [121,122,123]
PPAR-γ variant Pro12Ala	Regulation of the immune response [134,135]	Variant protecting from CD [134,135]
miR-595 and miR-1246	Small non-coding RNA molecule promote RNA silencing and post-transcriptional regulation of gene expression [139,140,141]	High levels of circulating miR-595 and miR-1246 are associated with a more aggressive form of the disease [141]
Polymorphisms of the gene BCMO1 (R267S: rs12934922 or A379V: rs7501331)	The conversion from beta-carotene to retinol does not occur [136,137,138]	Limited vitamin A production [136,137,138]
FFAR2, FADS1, FADS2, PPARA, PPARG, XRCC1, SCD1 gene mutations	Act on serum levels of LC-PUFA-omega-3 and omega-6 fatty acids [138]	Affect metabolic pathways and inflammation; increase cancer risk [138]
FUT2 gene loss of function	FUT2 function is lost [150]	Significant reduction of *Bifidobacterium bifidum*, *B. adolescentis*, and *B. catenulatum*/*pseudocatenulatum* colonization [150]

Abbreviations: IBD, Inflammatory Bowel Disease; PPAR-γ Peroxisome proliferator-activated receptor gamma; miR-595, MicroRNA 595; miR-1246, MicroRNA 1246; BCMO1, β,β-carotene-15,15’-monooxygenase 1; FFAR2, Free Fatty Acid Receptor 2; FADS1, Fatty Acid Desaturase 1; FADS2, Fatty Acid Desaturase 2; PPARA, Peroxisome Proliferator Activated Receptor Alpha; PPARG, Peroxisome Proliferator-Activated Receptor Gamma; XRCC1, X-ray Repair Cross Complementing 1; SCD1, Stearoyl-CoA Desaturase-1; LC-PUFA-omega-3 and omega-6, Long Chain Polyunsaturated Fatty Acids-omega-3 and omega-6; FUT2, Fucosyltransferase 2.
